# Serum lipids mediate the association of per- and polyfluoroalkyl substances exposure and age-related macular degeneration

**DOI:** 10.1371/journal.pone.0317678

**Published:** 2025-01-31

**Authors:** Xiaodong Chen, Jiaqi Li, Ningda Xu, Xuewei Li, Jiarui Li, Qianwen Guo, Jia Zhang, Heng Miao, Lvzhen Huang

**Affiliations:** 1 Department of Ophthalmology, Eye Diseases and Optometry Institute, Peking University People’s Hospital, Beijing, China; 2 Beijing Key Laboratory of Diagnosis and Therapy of Retinal and Choroid Diseases, Beijing, China; 3 College of Optometry, Peking University Health Science Center, Beijing, China; 4 College of Marxism, Capital Normal University, Beijing, China; 5 Shenzhen Eye Hospital, Guangdong, China; 6 Institute of Medical Technology, Peking University Health Science Center, Beijing, China; Texas A&M University College Station, UNITED STATES OF AMERICA

## Abstract

**Background:**

This study aims to investigate the connection between serum lipids, per- and polyfluoroalkyl substances (PFAS), and age-related macular degeneration (AMD) risk and assess whether serum lipids mediate the association between PFAS and AMD.

**Methods:**

1605 participants were enrolled from NHANES 2005–2008. Four serum PFAS levels with high detective rates, including perfluorononanoic acid (PFNA), perfluorooctanoic acid (PFOA), perfluorooctane sulfonic acid (PFOS), and perfluorohexane sulfonic acid (PFHxS) were examined. Restricted cubic spline analysis (RCS) and weighted quantile sum (WQS) analysis were employed to detect nonlinear and mixed exposure effects.

**Results:**

PFOS level was associated with any AMD (OR, 1.54; 95% CI, 1.12 to 2.11; P = 0.011), early AMD (OR, 1.43; 95% CI, 1.06 to 1.95; P = 0.024), and late AMD (OR, 3.35; 95% CI, 1.55 to 7.23; P = 0.004) risk. PFHxS (OR, 1.72; 95% CI, 1.01 to 2.93; P = 0.045) and PFOA (OR, 2.10; 95% CI, 1.21 to 3.63; P = 0.011) levels were associated with late AMD risk. The RCS showed a nonlinear connection between PFOS exposure and AMD risk (P nonlinear = 0.006). WQS analysis indicated a positive relationship between mixed PFAS exposure and AMD risk (OR, 1.34; 95% CI, 1.03 to 1.75; P = 0.030). Serum total cholesterol (TC) and high-density lipoprotein (HDL) cholesterol were associated with AMD risk (OR_TC_, 1.005; 95% CI, 1.001 to 1.009; P = 0.026. OR_HDL_, 1.028; 95% CI, 1.014 to 1.042; P<0.001), and mediated the relationship of PFOS exposure and AMD risk, with mediation proportions of 5.73% (P = 0.020) and 7.27% (P = 0.032), respectively.

**Conclusions:**

PFOS exposure was connected with AMD risk and serum TC and HDL mediated this relationship.

## 1. Introduction

Age-related macular degeneration (AMD) is an ocular neurodegenerative disease that leads to visual impairment and irreversibly vision loss, mainly affecting older adults [1]. Globally, 196 million patients suffered from AMD in 2020 [2]. Although the application of anti-vascular endothelial growth factor injections has made significant progression in the therapy of neovascular AMD (nAMD, a form of advanced AMD), many patients did not benefit much due to individual differences and no treatment was effective for early AMD [3, 4]. Besides, a patient with AMD treated with anti-VEGF therapy is expected to cost thousands of dollars per year [5]. AMD is considered to be caused by genetic and external factors [6]. Researchers have revealed many risk factors for AMD such as older age [7], tobacco smoking [8], diet [9], dyslipidemia [10], and cardiovascular diseases [11]. Currently, the effects of environmental pollutants exposure on AMD have become a research hotpot [12, 13].

Per- and polyfluoroalkyl substances (PFAS) are a group of synthetic fluorinated chemical substances [14]. PFAS are widely applied in plenty of industries, such as food packaging, textiles, and surfactants due to their properties, including stability, hydrophobicity, and oleophobic [15]. PFAS have a long biological half-life, resulting in a strong ability of bioaccumulation [16, 17]. The routes of PFAS exposure mainly include diet, indoor air, dust, and skin contact [18]. Serum PFAS levels were correlated with various human diseases such as kidney diseases, metabolic syndrome, and neurodegenerative diseases [19–21]. A study has revealed that perfluorooctane sulfonic acid (PFOS) was a biomarker for nAMD in a cohort with 46 nAMD patients who had undergone anti-VEGF therapy [22]. However, this study lacked the control group. A study has reported the relationship between PFAS exposure and ocular disorders, researchers found that high serum PFAS concentration was correlated with a higher prevalence of vision impairment and vitreous disorder in China [23]. Vitreous diseases are considered to be associated with AMD risk [24, 25]. Besides, it is reported that exposure to perfluorooctanoic acid (PFOA) could trigger inflammatory responses in retinal pigment epithelial cells [26], while inflammation engages in the development of AMD [27]. In addition, PFAS is widely reported to interfere with liver lipids metabolism and significantly increase the levels of serum lipids [28]. Drusen, the most distinguished histopathologic features of AMD, is mainly composed of lipids [29]. Altered lipid metabolism promotes the pathogenesis and development of AMD [10].

We aim to detect the connection between PFAS single and mixed exposure and AMD risk and perform a mediation analysis to assess whether serum lipids are involved in this relationship in a nationally representative cohort.

## 2. Methods

### 2.1 Participants

All data are sourced from the National Health and Nutrition Examination Survey (NHANES), and detailed information of NHANES is shown in its website [30]. The research involving human participants underwent a thorough review and received approval from the Research Ethics Review Board of the NCHS. All patients or participants gave written informed consent to be part of this study. This cross-sectional study started with the enrollment of 20497 participants from NHANES 2005–2006 and 2007–2008 cycles. After excluding those who lack data on the four PFAS (n = 16277) and examination of AMD (n = 2421), 1799 individuals remained. Then, participants with missing values on covariates were excluded (n = 194). Finally, 1605 individuals aged ≥40 were included (**S1 Fig**).

### 2.2 Fundus examination

Retinal images were captured in the NHANES 2005–2008 cycles using Canon CR6-45NM fundus imaging system [31, 32]. Early AMD: the presence of pigmentary abnormalities and/or soft drusen; late ARM: exudative ARM signs and/or geographic atrophy [33].

### 2.3 Assessment of PFAS

This study examined four highly detected PFAS compounds in the 2005–2008 NHANES cycle, including PFOA, perfluorohexane sulfonate (PFHxS), PFOS, and perfluorononanoic acid (PFNA). The results less than the low limits of detection (LLOD) were substituted as the LLOD value of each PFAS ⁄ square root of 2 [34].

### 2.4 Covariates

Carefully selected covariates were considered to control the potential effects of confounding factors: age, gender, education background, race, body mass index (BMI), serum high-density lipoprotein cholesterol levels (HDL, mg/dL), smoking (smoking at least 100 cigarettes in life were considered as smokers), family income-poverty ratio (PIR), drinking (having ≥ 12 alcohol drinks/year were defined as alcohol users), diabetes, history of cataract surgery, hypertension, and cardiovascular disease.

### 2.5 Statistical analysis

Following the guidelines for the complex oversampling data [35], we used subsample weights of PFAS (WTSA2YR in the 2005–2006 cycle and WTSC2YR in the 2007–2008 cycle). Characteristics of included individuals are recorded as means ± standard deviation (SD) or percentages. Data in skew distribution are reported as median ± interquartile range (IQR). Weighted logistic regression analyses were applied to determine 95% confidence intervals (CI) and odds ratios (OR). The logarithm-transformed PFAS (ln-PFAS) concentrations were added into the model as continuous variables, due to its non-normal distribution. The original PFAS serum concentration was also analyzed in tertiles. Crude model was not included any covariates; model 1 added age, sex, race, education level, family income-poverty ratio, and, BMI; model 2 additionally added serum HDL, alcohol drinking, hypertension, smoking, diabetes, history of cataract surgery, and cardiovascular diseases. We used restricted cubic spline curve (RCS) to find the nonlinear effect. The gWQS package of R software was employed to perform the weighted quantile sum regression (WQS) analysis for the four PFAS [36]. We divided 40% data into the test dataset and 60% into the validation dataset. Mediation analysis was done to assess whether the association of PFOS and AMD risk was mediated by serum lipids including low-density lipoprotein cholesterol (LDL), total cholesterol (TC), high-density lipoprotein cholesterol (HDL), and total triglyceride (TG). We achieved several sensitivity analyses. First, multiple imputation method was conducted to fill in the missing covariate data using R “mice” package [37]. Five imputed datasets were generated and one dataset was used for further analysis (sensitivity ⅰ). Second, we performed a sensitivity analysis by taking “age” as a categorical variable (<65 [n = 1052] or ≥65 [n = 553]) (sensitivity ⅱ). Third, we added serum cadmium into the model for further analysis (sensitivity ⅲ). All code of analyses were written with R 4.3.2.

## 3. Results

### 3.1 Study population

This study enrolled 1605 individuals. Among them, 114 participants were diagnosed with AMD (7.1%), of which 100 were early AMD and 14 were late AMD. 51.8% were females and 77.6% were non-Hispanic White. The average age was 56.0 years. Significant differences in age, race, family income-poverty ratio, serum HDL level, history of cataract surgery, and cardiovascular diseases were found between the two groups (P<0.05). Detailed information was shown in **Table 1**.

**Table 1:** Characteristics of included participants.

	All (n = 1605)	No AMD (n = 1491)	All AMD (n = 114)	P-value
Age, Mean (SD), y	56.0 (11.7)	55.3 (11.2)	67.4 (13.1)	<0.001
Sex, No. (%)				0.513
Male	826 (48.2)	764 (47.9)	62 (51.6)	
Female	779 (51.8)	727 (52.1)	52 (48.4)	
Race, No. (%)				<0.001
Non-Hispanic White	889 (77.6)	805 (76.9)	84 (89.8)	
Non-Hispanic Black	307 (8.9)	298 (9.3)	9 (2.5)	
Others	409 (13.5)	388 (13.8)	21 (7.7)	
BMI, kg/m^2^				0.053
<25	429 (28.3)	398 (28.7)	31 (22.1)	
25–30	575 (35.6)	523 (34.8)	52 (48.4)	
≥30	601 (36.1)	570 (36.5)	31 (29.6)	
Family income-poverty ratio	3.3 (1.6)	3.3 (1.6)	2.9 (1.5)	0.028
Education level, No. (%)				0.729
High school and below	456 (18.7)	425 (18.5)	31 (21.8)	
High school graduate	381 (24.5)	352 (24.5)	29 (24.2)	
Above high school	768 (56.8)	714 (57.0)	54 (54.0)	
HDL, Mean (SD), mg/dl	54.2 (17.1)	53.9 (17.7)	59.9 (16.8)	0.002
Smoking, No. (%)	859 (51.5)	793 (51.0)	66 (59.1)	0.146
Alcohol drinking, No. (%)	1090 (72.3)	1011 (72.2)	79 (73.7)	0.800
Hypertension, No. (%)	697 (39.5)	643 (39.3)	54 (43.4)	0.541
Diabetes, No. (%)	235 (11.3)	224 (11.3)	11 (10.8)	0.878
History of cataract surgery, No. (%)	201 (9.6)	167 (8.5)	34 (27.4)	<0.001
Cardiovascular diseases, No. (%)	230 (11.0)	192 (9.8)	38 (29.7)	<0.001

BMI, body mass index; PFHxS, perfluorohexane sulfonate; PFNA, per fluorononanoic acid; PFOA, perfluorooctanoic acid; PFOS, perfluorooctane sulfonic acid

### 3.2 Exposure

Compared to those individuals without AMD, AMD patients (especially late AMD) showed higher serum PFHxS and PFOS concentration (P<0.05) (**S1 Table**). A significantly positive correlations among the four original PFAS concentrations were detected (**S2 Fig**). PFNA, PFOA, and PFOS were strongly correlated in pairs (r>0.6).

### 3.3 PFAS exposure and AMD risk

**Table 2** showed the connection of PFAS and AMD risk. After adjusting for all covariates, ln-transformed PFOS level was connected with a higher risk of any AMD (OR, 1.54; 95% CI, 1.12 to 2.11; P = 0.011). Compared to the first tertile, the third tertile of PFOS showed an increased risk of any AMD (OR, 2.34; 95% CI, 1.27 to 4.33; P = 0.010).

**Table 2:** Logistic regression analysis of the association between PFAS exposure and AMD.

	Crude (OR, 95%CI)	P-value	Model 1 (OR, 95%CI)	P-value	Model 2 (OR, 95%CI)	P-value
PFHxS						
ln-PFHxS	**1.31 (1.09, 1.59)**	**0.006**	1.16 (0.93, 1.42)	0.164	1.16 (0.92, 1.45)	0.192
Q1 (<1.3 ng/mL)	Reference					
Q2 (1.3–2.7 ng/mL)	2.44 (0.89, 6.72)	0.082	2.11 (0.79, 5.66)	0.130	1.98 (0.65, 6.00)	0.210
Q3 (≥2.7 ng/mL)	**2.45 (1.22, 4.90)**	**0.013**	1.84 (0.96, 3.54)	0.065	1.77 (0.81, 3.85)	0.139
PFNA						
ln-PFNA	1.10 (0.77, 1.56)	0.587	1.09 (0.74, 1.60)	0.662	1.15 (0.77, 1.70)	0.473
Q1 (<0.902 ng/mL)	Reference					
Q2 (0.902–1.558 ng/mL)	1.44 (0.80, 2.58)	0.215	1.49 (0.82, 2.73)	0.183	1.73 (0.91, 3.29)	0.091
Q3 (≥1.558 ng/mL)	1.34 (0.70, 2.58)	0.364	1.31 (0.63, 2.71)	0.449	1.48 (0.69, 3.17)	0.285
PFOA						
ln-PFOA	1.30 (0.89, 1.90)	0.173	1.11 (0.75, 1.65)	0.587	1.23 (0.79, 1.92)	0.336
Q1 (<3.5 ng/mL)	Reference					
Q2 (3.5–5.6 ng/mL)	1.39 (0.89, 2.18)	0.140	1.18 (0.74, 1.88)	0.472	1.29 (0.76, 2.20)	0.325
Q3 (≥5.6 ng/mL)	1.57 (0.84, 2.93)	0.148	1.28 (0.69, 2.37)	0.420	1.45 (0.76, 2.78)	0.243
PFOS						
ln-PFOS	**1.77 (1.30, 2.41)**	**<0.001**	**1.43 (1.04, 1.96)**	**0.028**	**1.54 (1.12, 2.11)**	**0.011**
Q1 (<13.2 ng/mL)	Reference					
Q2 (13.2–24.3 ng/mL)	2.04 (0.89, 4.64)	0.088	1.66 (0.73, 3.79)	0.217	1.91 (0.80, 4.55)	0.133
Q3 (≥24.3 ng/mL)	**3.11 (1.66, 5.84)**	**<0.001**	**2.00 (1.07, 3.75)**	**0.031**	**2.34 (1.27, 4.33)**	**0.010**

PFAS: perfluoroalkyl substances; PFHxS, perfluorohexane sulfonate; PFNA, per fluorononanoic acid; PFOA, perfluorooctanoic acid; PFOS, perfluorooctane sulfonic acid

The crude model was adjusted for None.

Model 1 was adjusted for age, sex, race, education level, family income-poverty ratio, and, BMI.

Model 2 was adjusted for age, sex, race, education level, family income-poverty ratio, BMI, serum HDL, smoking, alcohol drinking, hypertension, diabetes, history of cataract surgery, and cardiovascular diseases.

**Table 3** showed the connection between PFAS and different AMD type risk. Ln-transformed PFOS level was significantly associated with a higher risk of early AMD (OR, 1.43; 95% CI, 1.06 to 1.95; P = 0.024), and ln-transformed PFHxS (OR, 1.72; 95% CI, 1.01 to 2.93; P = 0.045), PFOA (OR, 2.10; 95% CI, 1.21 to 3.63; P = 0.011), and PFOS (OR, 3.35; 95% CI, 1.55 to 7.23; P = 0.004) levels were connected with a higher late AMD risk.

**Table 3:** The association between PFAS exposure and early and late AMD.

	Crude (OR, 95%CI)	P-value	Model 1 (OR, 95%CI)	P-value	Model 2 (OR, 95%CI)	P-value
Early AMD						
ln-PFHxS	**1.28 (1.05, 1.57)**	**0.017**	1.14 (0.91, 1.41)	0.240	1.14 (0.90, 1.44)	0.258
ln-PFNA	1.15 (0.78, 1.69)	0.481	1.12 (0.75, 1.67)	0.567	1.20 (0.79, 1.81)	0.363
ln-PFOA	1.23 (0.83, 1.83)	0.284	1.06 (0.71, 1.57)	0.763	1.19 (0.75, 1.90)	0.442
ln-PFOS	**1.63 (1.19, 2.21)**	**0.003**	1.33 (0.98, 1.79)	0.063	**1.43 (1.06, 1.95)**	**0.024**
Late AMD						
ln-PFHxS	**1.56 (1.01, 2.41)**	**0.047**	1.59 (0.91, 2.79)	0.098	**1.72 (1.01, 2.93)**	**0.045**
ln-PFNA	0.81 (0.45, 1.46)	0.464	0.85 (0.46, 1.58)	0.594	0.90 (0.45, 1.80)	0.755
ln-PFOA	**1.92 (1.14, 3.24)**	**0.016**	**2.09 (1.12, 3.90)**	**0.022**	**2.10 (1.21, 3.63)**	**0.011**
ln-PFOS	**3.22 (1.97, 5.27)**	**<0.001**	**3.35 (1.52, 7.39)**	**0.004**	**3.35 (1.55, 7.23)**	**0.004**

PFAS: perfluoroalkyl substances; PFHxS, perfluorohexane sulfonate; PFNA, per fluorononanoic acid; PFOA, perfluorooctanoic acid; PFOS, perfluorooctane sulfonic acid

The crude model was adjusted for None.

Model 1 was adjusted for age, sex, race, education level, family income-poverty ratio, and, BMI.

Model 2 was adjusted for age, sex, race, education level, family income-poverty ratio, BMI, serum HDL, smoking, alcohol drinking, hypertension, diabetes, history of cataract surgery, and cardiovascular diseases.

**Fig 1:**
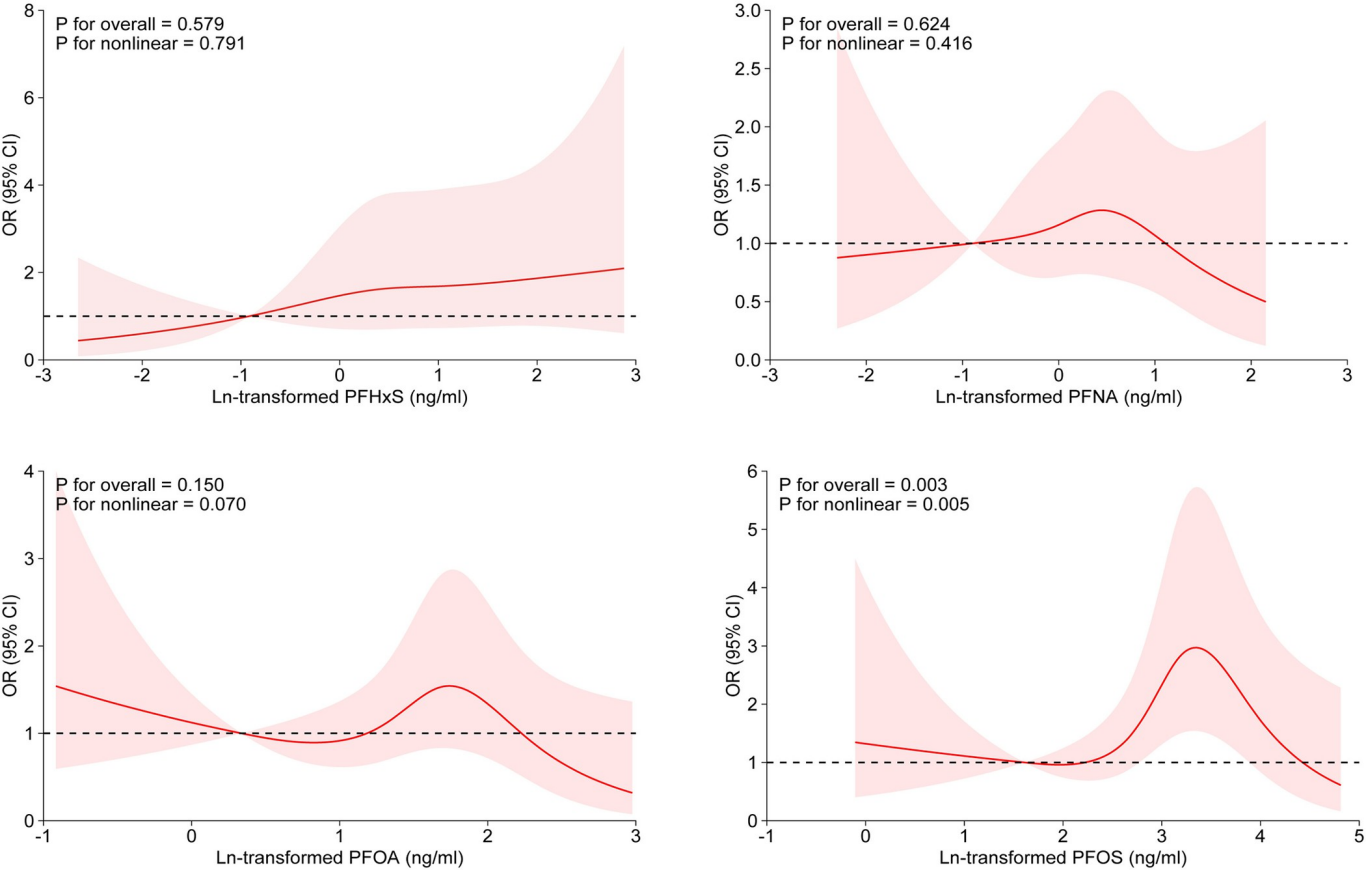
Dose-response relationship between natural logarithm-transformed transformed serum per- and polyfluoroalkyl substances (PFAS) concentrations and age-related macular degeneration. PFOA, perfluorooctanoic acid; PFOS, perfluorooctane sulfonic acid; PFHxS, perfluorohexane sulfonic acid; PFNA, perfluorononanoic acid. The model was adjusted for age, sex, race, education level, family income-poverty ratio, BMI, serum HDL, smoking, alcohol drinking, hypertension, diabetes, history of cataract surgery, and cardiovascular diseases.

Fig 1 showed the RCS curve of each PFAS exposure and AMD risk. A nonlinear association was found between ln-transformed PFOS level and the risk of AMD (P for nonlinearity = 0.006).

### 3.4 WQS analysis

WQS analysis indicated a significant positive relationship between mixed PFAS exposure and AMD risk (OR, 1.34; 95% CI, 1.03 to 1.75; P = 0.030). PFOS accounted for 93.9% of total weights (**S3 Fig**).

### 3.5 Subgroup analysis

After stratified by sex, ln-transformed PFOS level was connected with a higher risk of AMD in men (OR, 2.00; 95% CI, 1.26 to 3.18; P = 0.006). After grouping by hypertension, ln-transformed PFNA (OR, 1.49; 95% CI, 1.02 to 2.19; P = 0.042), PFOA (OR, 2.34; 95% CI, 1.22 to 4.49; P = 0.014), and PFOS (OR, 2.09; 95% CI, 1.45 to 3.04; P<0.001) levels were connected with AMD risk in those who suffered from hypertension. Besides, PFOS exposure was connected with AMD risk in those who were smokers (OR, 1.82; 95% CI, 1.28 to 2.59; P = 0.002), drinkers (OR, 1.79; 95% CI, 1.24 to 2.58; P = 0.004), and had cardiovascular diseases (OR, 1.88; 95% CI, 1.13 to 3.14; P = 0.020) (**S2 Table**).

### 3.6 Mediation analysis

After adding all covariates in the model, serum TC (OR, 1.005; 95% CI, 1.001 to 1.009; P = 0.026) and HDL (OR, 1.028; 95% CI, 1.014 to 1.042; P<0.001) levels were connected with AMD risk (**S3 Table**). Next, we performed a mediation analysis, which showed that serum TC and HDL levels significantly mediated the connection between PFOS exposure and AMD risk and the mediated proportions were 5.73% (P = 0.020) and 7.27% (P = 0.032), respectively, while no significant mediation effects were detected for LDL-C and TG (P > 0.05) (**Table 4**).

**Table 4:** The mediating effects of lipids on the association between logarithm-transformed PFOS and AMD risk.

Lipids	Participants (n)	Direct effects	Indirect effects	Total effects	Mediated proportion (%)	P-value
		β (95%CI)	β (95%CI)	β (95%CI)		
TC (mg/dL)	1605	0.0090 (0.0064, 0.0119) ***	0.0007 (0.0001, 0.0019) *	0.0097 (0.0074, 0,0125) ***	5.73 %	**0.020**
HDL (mg/dL)	1605	0.0088 (0.0064, 0.0117) ***	0.0008 (0.0001, 0.0020) *	0.0095 (0.0072, 0.0125) ***	7.27 %	**0.032**
LDL (mg/dL)	752	0.0123 (0.0073, 0.0183) ***	0.0001 (-0.0014, 0.0014)	0.0125 (0.0075, 0.0184) ***	0.86%	0.728
TG (mg/dL)	752	0.0125 (0.0072, 0.0181) ***	0.0001 (-0.0010, 0.0013)	0.0126 (0.0076, 0.0182) ***	0.57%	0.752

Abbreviations: TC: Total cholesterol; HDL: High-density lipoprotein cholesterol; LDL: Low-density lipoprotein cholesterol; TG: Total triglyceride; CI, confidence interval; PFOS, perfluorooctane sulfonic acid.

Model was adjusted for age, sex, race, education level, family income-poverty ratio, BMI, smoking, alcohol drinking, self-reported health, hypertension, diabetes, history of cataract surgery, and cardiovascular diseases.

*P<0.05

**P<0.01

***P<0.001

### 3.7 Sensitivity analyses

We used sensitivity analyses to validate our results (**S4 Table**), which showed comparable findings.

## 4. Discussion

This study indicated that PFOS exposure was connected with a higher risk of early, late, and any AMD, and exposure to PFHxS and PFOA was connected with a higher late AMD risk. Serum TC and HDL involved in the effects of PFOS on AMD risk. A nonlinear relationship was detected between PFOS exposure and AMD risk. WQS analysis displayed that PFOS had the most positive weight in the mixed PFAS exposure.

An isomer of the C8 Health Project in China found no significant connection between PFAS exposure and macular disorder and retinal disorder, researchers pointed out this might be attributed to the low prevalence such as macular disorder (2.3%) [23]. It was noteworthy that authors only observed ocular conditions (such as pigment disorder in the macular area) but did not evaluate the specific disease (such as AMD). In our study, AMD was diagnosed by two retinal specialists, with a prevalence of 7.1%. Besides, PFOS exposure showed the strongest association with AMD risk. Many studies reported that PFOS exposure was strongly associated with the prevalence of diseases compared to other PFAS monomers [38–40]. An experimental study revealed that PFOS showed the strongest ability of DNA damage to the HepG2 cell line among seven PFAS, including PFHxS, PFNA, PFOA, PFOS [41].

In the subgroup analysis, a positive relationship between PFOS exposure and AMD was observed in males. Compared to females, the four serum PFAS levels were higher in males (P < 0.001, data not shown) in this study. This may be attributed to the differences in exposure patterns metabolism or kinetics [42]. Higher concentrations may reach the threshold of inducing diseases. Besides, PFOS exposure was positively correlated with AMD risk, especially in those participants with risk factors of AMD, including smoking, alcohol drinking, hypertension, diabetes, and cardiovascular diseases [43, 44].

A recent study indicated that exposure to PFOS can cause significant apoptosis using the retinal pre-organoid model [45]. In addition, one study reported that PFOS exposure significantly reduced the expression of most genes that participated in the opsins in the process of the phototransduction cascade, leading to vision impairment [46]. PFOS could also cause the death of photoreceptors (661W) by upregulating inflammatory response [47]. Romano et al. have found that the cytotoxicity limit of PFOA on ARPE-19 cells was 28.4 ppm [48]. Recently, plenty of studies have provided evidence of the toxicity of PFOA exposure to ocular cells [48–50]. In this present study, PFOA was linearly associated with late AMD risk. The potential molecular mechanism is unclear. Given the lack of evidence, we hypothesized that several PFAS exposure-induced pathophysiological processes may participate in the onset and development of AMD, including lipid deposition, oxidative stress, and chronic inflammation. First, plenty of evidence has confirmed that exposure to PFAS could increase the risk of hepatic steatosis [51, 52] and dyslipidemia [53]. PFOS was significantly connected with higher serum lipids levels [28]. Lipids are a main component of drusen, and retinal pigment epithelium (RPE) cells can accumulate cholesterol from lipoproteins in the circulation and also can recycle it to the photoreceptors or eliminate it by the formation of high-density lipoprotein particles [54]. When some factors such as dyslipidemia disorder the process of reverse cholesterol transport, lipoprotein particles complex may deposit in the Bruch’s Membrane and RPE [55]. Thus, exposure to PFAS may accumulate lipids in the retina or plasma and involve the development of AMD. Second, the retina continuously converts light into electrical signals that require lots of energy and generate reactive oxygen species (ROS) [56]. Nevertheless, excessive ROS will trigger oxidative stress, causing the damage of cellular proteins, DNA, lipids, and impairing the structure and function of the retina [57]. Oxidative stress may be the central process in the onset and development of AMD [58]. PFAS has been reported strong ability to induce oxidative stress by upregulating multiple biomarkers [59, 60]. Third, some researchers propose that the combined factors of immune triggers and the resulting inflammatory response in the pathogenesis of AMD (also known as a two-level model hypothesis) [61]. In 2016, the US National Toxicology Program evaluated and reported PFOA and PFOS exposure-induced immunotoxicity [62]. PFAS has been widely reported to affect inflammation in various organs, such as the lungs, intestines, and liver [63–65]. Thus, PFAS is likely to induce inflammation and then influence the development of AMD. Our results showed that High TC and HDL were connected with a higher AMD risk, and TC and HDL significantly mediated the effects of PFOS on AMD risk. It is reported that a high-cholesterol diet can cause age-related macular degeneration-like pathology in rats [66]. In addition, high serum HDL was widely considered to have a causal relationship with an increased AMD risk [67]. More studies are required to explore the above and other potential mechanisms further in the future.

This current study had some advantages, including prospective strong national representativeness of the study population and multiple statistical methods consisting of logistical regression, RCS curve, WQS analysis, and mediation analysis, which can fully understand the connection between PFAS and AMD. Nevertheless, several limitations existed that should be considered rigorously when interpreting these findings. First, the causation could not be determined. Second, we only examined four highly detected PFAS, and other PFAS did not be analyzed. Third, though multiple covariates were adjusted in our model, the effects of other confounding factors, such as vitreoretinal surgery and genetic factors, could not be considered completely.

## 5. Conclusion

This study indicates that PFOS was strongly connected with a higher early and late AMD risk, and PFOA and PFHxS were connected with late AMD risk. Serum TC and HDL mediate the connection of PFOS and AMD risk. Further longitudinal studies are required to verify these findings.

## Supporting information

S1 Fig Selection of study population. (DOCX)

S2 Fig Correlation analysis. PFOA, perfluorooctanoic acid; PFOS, perfluorooctane sulfonic acid; PFHxS, perfluorohexane sulfonic acid; PFNA, perfluorononanoic acid.
(DOCX)

S3 Fig Contribution of PFAS to WQS percentage for age-related macular degeneration. PFOA, perfluorooctanoic acid; PFOS, perfluorooctane sulfonic acid; PFHxS, perfluorohexane sulfonic acid; PFNA, perfluorononanoic acid.
(DOCX)

S1 Table Serum concentrations of PFAS among groups. (DOCX)

S2 Table Subgroup analysis. (DOCX)

S3 Table The association between serum lipids and AMD risk. (DOCX)

S4 Table Sensitivity analysis. (DOCX)
