# Expression of Concern: Serum lipids mediate the association of per- and polyfluoroalkyl substances exposure and age-related macular degeneration

**DOI:** 10.1371/journal.pone.0355440

**Published:** 2026-08-06

**Authors:** The PLOS One Editors

After this article [1] was published, the *PLOS One* Editors noted concerns about the article’s peer review and compliance with the PLOS Authorship policy.

In light of the cumulative issues, the *PLOS One* Editors issue this Expression of Concern. Readers are advised to interpret the article [1] with caution.

The Editors do not have concerns regarding the authors’ conduct during the peer review process.
