# Supplementary material for: Serum lipids mediate the association of per- and polyfluoroalkyl substances exposure and age-related macular degeneration
Source: PLoS One. 2025 Jan 31;20(1):e0317678. doi: 10.1371/journal.pone.0317678 (PMC11785341; doi:10.1371/journal.pone.0317678)
Supplement: S1 Fig — (DOCX) [file pone.0317678.s001.docx]

n = 1605

n = 1799

Excluded participants without examination of prevalence of AMD

(n = 2421)

(n = 698)

Excluded participants without covariates

(n = 194)

Participants from NHANES 2005-2008

n = 20497

n = 4220

Excluded participants without data on perfluoroalkyl substances

(n = 16277)

**S1 Figure. Selection of study population.**
