# Supplementary material for: Serum lipids mediate the association of per- and polyfluoroalkyl substances exposure and age-related macular degeneration
Source: PLoS One. 2025 Jan 31;20(1):e0317678. doi: 10.1371/journal.pone.0317678 (PMC11785341; doi:10.1371/journal.pone.0317678)
Supplement: S2 Fig — PFOA, perfluorooctanoic acid; PFOS, perfluorooctane sulfonic acid; PFHxS, perfluorohexane sulfonic acid; PFNA, perfluorononanoic acid. (DOCX) [file pone.0317678.s002.docx]

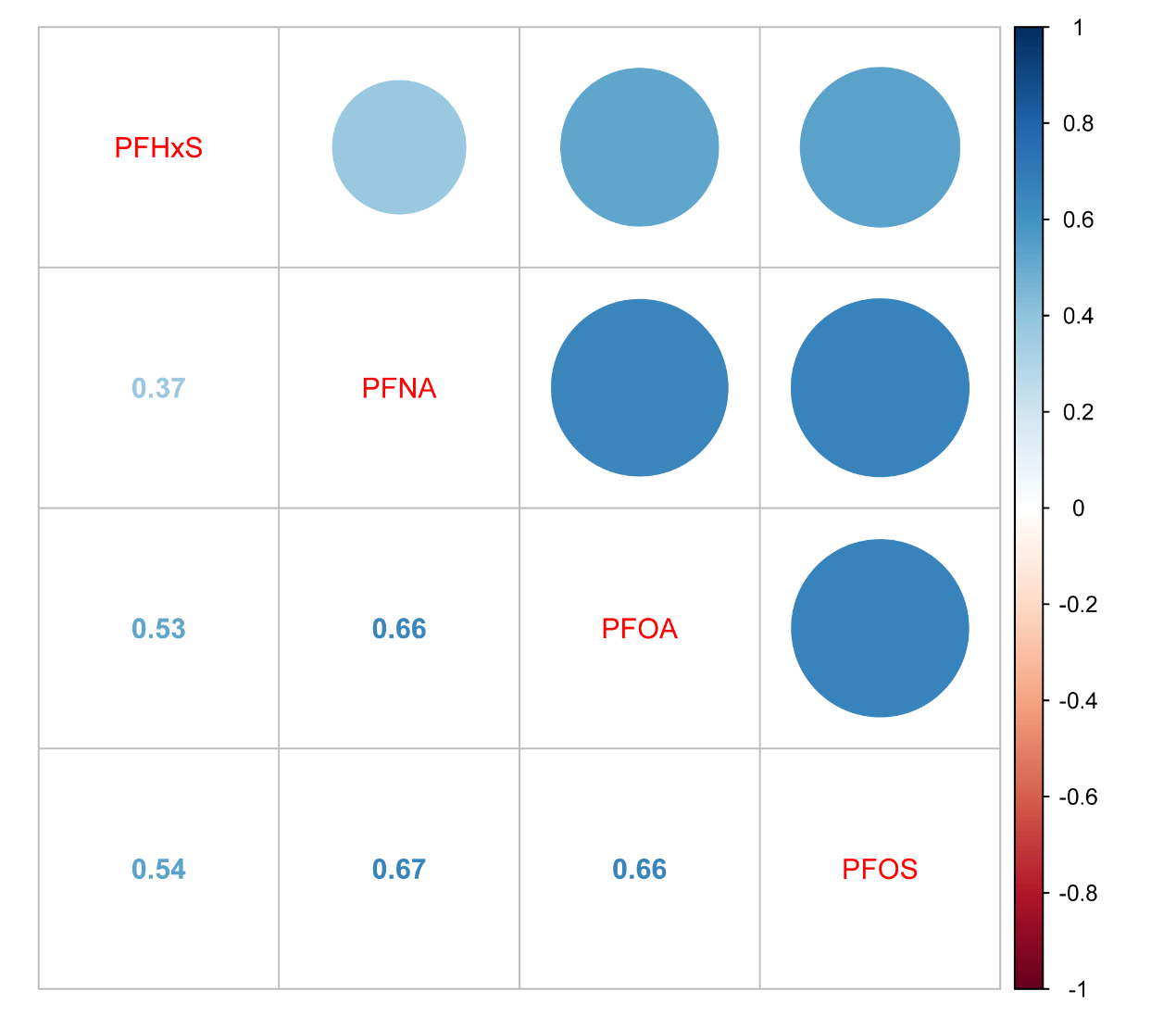
PFOA, perfluorooctanoic acid; PFOS, perfluorooctane sulfonic acid; PFHxS, perfluorohexane sulfonic acid; PFNA, perfluorononanoic acid.

**S2 Figure. Correlation analysis.**
