# Supplementary material for: Serum lipids mediate the association of per- and polyfluoroalkyl substances exposure and age-related macular degeneration
Source: PLoS One. 2025 Jan 31;20(1):e0317678. doi: 10.1371/journal.pone.0317678 (PMC11785341; doi:10.1371/journal.pone.0317678)
Supplement: S1 Table — (DOCX) [file pone.0317678.s004.docx]

**S1 Table. Serum concentrations of PFAS among groups.**

|  | Non-AMD (n=1491) | All AMD  (n=114) | Early AMD (n=100) | Late AMD  (n=14) | P-Value |
| --- | --- | --- | --- | --- | --- |
| PFHxS, Median (IQR), ng/ml | 1.9 (2.2) | 2.2 (1.8) | 2.0 (1.8) | 2.9 (1.8) | **0.017** |
| PFNA, Median (IQR), ng/ml | 1.2 (1.0) | 1.3 (0.9) | 1.3 (0.8) | 1.0 (1.3) | 0.463 |
| PFOA, Median (IQR), ng/ml | 4.5 (3.4) | 4.6 (3.6) | 4.4 (3.6) | 5.1 (3.7) | 0.172 |
| PFOS, Median (IQR), ng/ml | 17.4 (17.0) | 23.4 (16.1) | 23.0 (14.8) | 29.6 (22.1) | **<0.001** |

PFAS: perfluoroalkyl substances; PFHxS, perfluorohexane sulfonate; PFNA, per fluorononanoic acid; PFOA, perfluorooctanoic acid; PFOS, perfluorooctane sulfonic acid

The P-Value was computed from the comparison between the Non-AMD and all AMD groups.
