# Supplementary material for: Serum lipids mediate the association of per- and polyfluoroalkyl substances exposure and age-related macular degeneration
Source: PLoS One. 2025 Jan 31;20(1):e0317678. doi: 10.1371/journal.pone.0317678 (PMC11785341; doi:10.1371/journal.pone.0317678)
Supplement: S2 Table — (DOCX) [file pone.0317678.s005.docx]

**S2 Table. Subgroup analysis**

| PFAS exposure (ng/ml) | Ln-transformed PFHxS | Ln-transformed PFNA | Ln-transformed PFOA | Ln-transformed PFOS |
| --- | --- | --- | --- | --- |
| Sex |  |  |  |  |
| Male | 0.90 (0.69, 1.19); 0.436 | 1.60 (0.92, 2.77); 0.089 | 1.43 (0.80, 2.56); 0.208 | **2.00 (1.26, 3.18); 0.006** |
| Female | 1.28 (0.90, 1.84); 0.159 | 0.87 (0.58, 1.30); 0.467 | 0.97 (0.54, 1.74); 0.905 | 1.24 (0.88, 1.75); 0.207 |
| Smoking |  |  |  |  |
| Yes | 1.08 (0.77, 1.53); 0.634 | 1.31 (0.87, 1.98); 0.181 | 1.56 (0.91, 2.66); 0.096 | **1.82 (1.28, 2.59); 0.002** |
| No | 1.21 (0.84, 1.76); 0.280 | 1.09 (0.65, 1.83); 0.718 | 0.76 (0.47, 1.22); 0.241 | 1.32 (0.84, 2.07); 0.206 |
| Alcohol drinking |  |  |  |  |
| Yes | 1.13 (0.84, 1.51); 0.410 | 1.19 (0.82, 1.74); 0.339 | 1.45 (0.87, 2.41); 0.141 | **1.79 (1.24, 2.58); 0.004** |
| No | 1.13 (070, 1.84); 0.592 | 1.29 (0.69, 2.43); 0.406 | 0.80 (0.46, 1.39); 0.409 | 1.44 (0.82, 2.52); 0.188 |
| Hypertension |  |  |  |  |
| Yes | 1.43 (0.96, 2.13); 0.077 | **1.49 (1.02, 2.19); 0.042** | **2.34 (1.22, 4.49); 0.014** | **2.09 (1.45, 3.04); <0.001** |
| No | 1.01 (0.78, 1.32); 0.920 | 0.97 (0.56, 1.70); 0.917 | 0.84 (0.52, 1.35); 0.440 | 1.31 (0.91, 1.87); 0.135 |
| Diabetes |  |  |  |  |
| Yes | 1.03 (0.53, 2.01); 0.928 | 2.08 (0.68, 6.40); 0.184 | **2.79 (1.21, 6.39); 0.019** | **3.33 (1.52, 7.34); 0.005** |
| No | 1.18 (0.93, 1.49); 0.166 | 1.09 (0.71, 1.67); 0.669 | 1.12 (0.71, 1.76); 0.598 | **1.46 (1.04, 2.07); 0.032** |
| Cardiovascular diseases |  |  |  |  |
| Yes | 1.94 (0.96, 3.79); 0.051 | 2.40 (0.91, 6.29); 0.071 | 2.65 (0.89, 7.90); 0.075 | **1.88 (1.13, 3.14); 0.020** |
| No | 1.06 (0.86, 1.30); 0.569 | 0.87 (0.56, 1.36); 0.514 | 0.99 (0.62, 1.59); 0.984 | 1.40 (0.95, 2.07); 0.086 |

PFAS: perfluoroalkyl substances; PFHxS, perfluorohexane sulfonate; PFNA, per fluorononanoic acid; PFOA, perfluorooctanoic acid; PFOS, perfluorooctane sulfonic acid.

Data was presented in OR 95%CI; P-value.

Subgroup analysis was performed based on the Model 2, which was adjusted for age, sex, race, education level, family income-poverty ratio, BMI, serum HDL, smoking, alcohol drinking, hypertension, diabetes, history of cataract surgery, and cardiovascular diseases.
