# Supplementary material for: Serum lipids mediate the association of per- and polyfluoroalkyl substances exposure and age-related macular degeneration
Source: PLoS One. 2025 Jan 31;20(1):e0317678. doi: 10.1371/journal.pone.0317678 (PMC11785341; doi:10.1371/journal.pone.0317678)
Supplement: S3 Table — (DOCX) [file pone.0317678.s006.docx]

**S3 Table. The association between serum lipids and AMD risk.**

|  | Participants (n) | Crude  (OR, 95%CI) | P-value | Model 1  (OR, 95%CI) | P-value | Model 2  (OR, 95%CI) | P-value |
| --- | --- | --- | --- | --- | --- | --- | --- |
| TC (mg/dL) | 1605 | 1.002 (0.996, 1.008) | 0.458 | 1.004 (0.997, 1.009) | 0.066 | **1.005 (1.001, 1.009)** | **0.026** |
| HDL (mg/dL) | 1605 | **1.018 (1.008, 1.028)** | **<0.001** | **1.028 (1.015, 1.041)** | **<0.001** | **1.028 (1.014, 1.042)** | **<0.001** |
| LDL (mg/dL) | 752 | 1.003 (0.994, 1.011) | 0.564 | 1.005 (0.999, 1.012) | 0.114 | 1.006 (0.999, 1.012) | 0.079 |
| TG (mg/dL) | 752 | 1.000 (0.995, 1.004) | 0.891 | 0.998 (0.991, 1.004) | 0.473 | 0.998 (0.991, 1.004) | 0.456 |

PFAS: perfluoroalkyl substances; PFHxS, perfluorohexane sulfonate; PFNA, per fluorononanoic acid; PFOA, perfluorooctanoic acid; PFOS, perfluorooctane sulfonic acid; TC: Total cholesterol; HDL: High-density lipoprotein cholesterol; LDL: Low-density lipoprotein cholesterol; TG: Total triglyceride; CI, confidence interval.

Crude model was adjusted for None.

Model 1 was adjusted for age, sex, race, education level, family income-poverty ratio, and, BMI.

Model 2 was adjusted for age, sex, race, education level, family income-poverty ratio, BMI, smoking, alcohol drinking, hypertension, diabetes, history of cataract surgery, and cardiovascular diseases.
