# Supplementary material for: Serum lipids mediate the association of per- and polyfluoroalkyl substances exposure and age-related macular degeneration
Source: PLoS One. 2025 Jan 31;20(1):e0317678. doi: 10.1371/journal.pone.0317678 (PMC11785341; doi:10.1371/journal.pone.0317678)
Supplement: S4 Table — (DOCX) [file pone.0317678.s007.docx]

**S4 Table. Sensitivity analysis.**

|  | Sensitivity ⅰ | |  | Sensitivity ⅱ | |  | Sensitivity ⅲ | |
| --- | --- | --- | --- | --- | --- | --- | --- | --- |
|  | OR (95%CI) | P-value |  | OR (95%CI) | P-value |  | OR (95%CI) | P-value |
| Ln-transformed PFHxS (ng/ml) |  |  |  |  |  |  |  |  |
| Continuous | 1.12 (0.92, 1.36) | 0.232 |  | 1.21 (0.97, 1.51) | 0.090 |  | 1.16 (0.92, 1.46) | 0.182 |
| Tertile 1 | Reference | |  | Reference | |  | Reference | |
| Tertile 2 | 1.18 (0.49, 2.83) | 0.690 |  | 2.02 (0.67, 6.08) | 0.194 |  | 1.97 (0.64, 6.05) | 0.214 |
| Tertile 3 | 1.35 (0.77, 2.35) | 0.272 |  | 1.96 (0.91, 4.22) | 0.079 |  | 1.72 (0.86, 3.43) | 0.138 |
| Ln-transformed PFNA (ng/ml) |  |  |  |  |  |  |  |  |
| Continuous | 1.20 (0.84, 1.70) | 0.298 |  | 1.17 (0.81, 1.70) | 0.379 |  | 1.14 (0.76, 1.71) | 0.507 |
| Tertile 1 | Reference | |  | Reference | |  | Reference | |
| Tertile 2 | 1.57 (0.83, 2.95) | 0.152 |  | 1.86 (0.98, 3.56) | 0.058 |  | 1.72 (0.86, 3.43) | 0.112 |
| Tertile 3 | 1.47 (0.70, 3.12) | 0.286 |  | 1.54 (0.73, 3.24) | 0.230 |  | 1.46 (0.67, 3.20) | 0.310 |
| Ln-transformed PFOA (ng/ml) |  |  |  |  |  |  |  |  |
| Continuous | 1.19 (0.82, 1.73) | 0.324 |  | 1.26 (0.84, 1.88) | 0.240 |  | 1.24 (0.78, 1.95) | 0.333 |
| Tertile 1 | Reference | |  | Reference | |  | Reference | |
| Tertile 2 | 1.35 (0.81, 2.26) | 0.233 |  | 1.36 (0.77, 2.38) | 0.264 |  | 1.30 (0.75, 2.25) | 0.321 |
| Tertile 3 | 1.48 (0.82, 2.68) | 0.177 |  | 1.51 (0.81, 2.83) | 0.179 |  | 1.47 (0.75, 2.88) | 0.240 |
| Ln-transformed PFOS (ng/ml) |  |  |  |  |  |  |  |  |
| Continuous | **1.41 (1.06, 1.88)** | **0.020** |  | **1.63 (1.18, 2.26)** | **0.006** |  | **1.53 (1.11, 2.11)** | **0.013** |
| Tertile 1 | Reference | |  | Reference | |  | Reference | |
| Tertile 2 | 1.72 (0.74, 4.03) | 0.192 |  | 2.17 (0.89, 5.28) | 0.083 |  | 1.87 (0.78, 4.50) | 0.147 |
| Tertile 3 | **2.10 (1.14, 3.89)** | **0.021** |  | **2.65 (1.41, 5.00)** | **0.005** |  | **2.31 (1.25, 4.29)** | **0.012** |

PFAS: perfluoroalkyl substances; PFHxS, perfluorohexane sulfonate; PFNA, per fluorononanoic acid; PFOA, perfluorooctanoic acid; PFOS, perfluorooctane sulfonic acid

Model was adjusted for age, sex, race, education level, family income-poverty ratio, BMI, serum HDL, smoking, alcohol drinking, hypertension, diabetes, history of cataract surgery, and cardiovascular diseases. Sensitivity ⅲ was additionally adjusted for serum cadmium.
